# Association between hospital internal medicine physician workforce and patient admissions during the COVID-19 pandemic in Japan

**DOI:** 10.1186/s12913-023-09043-0

**Published:** 2023-01-21

**Authors:** Seiji Hamada, Takuhiro Moromizato, Masashi Narita, Kiyosu Taniguchi, Kenji Shibuya, Yasuharu Tokuda

**Affiliations:** 1 Urasoe General Hospital, Urasoe, Okinawa, Japan; 2 Okinawa Nanbu Medical Center, Haebaru, Okinawa, Japan; 3 The Tokyo Foundation for Policy Research, Minato-ku, Tokyo, Japan; 4 National Hospital Organization Mie National Hospital, Tsu, Japan

**Keywords:** Physician shortage, Physician staffing, Patient admission, Ambulance transport, COVID-19

## Abstract

**Background:**

Hospital physician workforce in Japan is the lowest among developed countries. Many patients with novel coronavirus disease 2019 (COVID-19) with high risk of mortality could not be hospitalized during case surges in Japan and only about 5% of total acute care beds were used as COVID-19 beds nationwide. However, the relationship between the number of hospital physicians and patient admissions remains unclear. Thus, we aimed to evaluate this relationship in areas with the highest incidences during the surges.

**Methods:**

Data collection was performed for teaching hospitals accredited with the specialty of internal medicine in three prefectures which experienced the highest COVID-19 incidences in Japan (Tokyo, Osaka, Okinawa). Association was examined between the number of full-time physicians (internal medicine staff physicians and residents) and admissions of internal medicine patients through ambulance transport from April 2020 to March 2021. Analysis was conducted separately for community hospitals and university hospitals because the latter have roles as research institutions in Japan. Community hospitals included private, public, and semi-public hospitals.

**Results:**

Of 117 teaching hospitals in three prefectures, data from 108 teaching hospitals (83 community hospitals and 25 university hospitals) were available. A total of 102,400 internal medicine patients were admitted to these hospitals during the one-year period. Private hospitals received the greatest mean number of patient admissions (290 per 100 beds), followed by public hospitals (227) and semi-public hospitals (201), and university hospitals (94). Among community hospitals, a higher number of resident physicians per 100 beds was significantly associated with a greater number of patient admissions per 100 beds with beta coefficient of 11.6 (95% CI, 1.5 to 21.2, *p* = 0.025) admissions by one physician increase per 100 beds. There was no such association among university hospitals.

**Conclusions:**

Community hospitals with many resident physicians accepted more internal medicine admissions through ambulance transport during the COVID-19 pandemic. An effective policy to counter physician shortage in hospitals in Japan may be to increase internal medicine resident physicians among community hospitals to save more lives.

## Background

During the COVID-19 pandemic, hospitals in many countries have faced massive patient admission surges through ambulance transport. Although Japan has the highest number of acute care beds per 1000 population compared to other developed countries [1], many COVID-19 patients with high risk of mortality could not be hospitalized. In fact, during a single month with the Delta variant wave in Japan, there were more than 200 patients who died at home while waiting for admission to hospitals [2]. In that month, only 71% of available beds (COVID-19 beds) were used even at the peak admission phase in Tokyo, the prefecture with the highest COVID-19 incidence [3]. Moreover, 2 years after the pandemic initiation, only about 5% (38,997 beds) of total acute care beds (897, 356 beds) were used as COVID-19 beds nationwide [4].

Ambulance transport in Japan is free of charge for patients and is run by local governments. During the COVID-19 pandemic, in accordance with the Infectious Disease Control Law, central and local governments have tried controlling patient flow for those with definite or suspected COVID-19 [5]. Basically, patients who require oxygen therapy are considered for hospital admission. Hospitals in Japan can refuse or accept patients for admission when ambulance emergency medical technicians call them by phone. Typical reasons for refusal are that acute care beds are full of current inpatients or there is an inadequacy of healthcare providers (physicians and/or nurses). During the pandemic, the Infectious Disease Control Law has been enacted for asking hospitals to maintain many beds for a possible surge of patient admissions. However, in Japan, infectious disease beds have been secured only voluntarily by most hospitals, and thus patients may be reallocated to other hospitals, or they may have to stay at home or in temporary medical facilities. Physician shortage in hospitals has been claimed as a major factor in the reduced acceptance of patient admission. The workforce of physicians working full-time in Japanese hospitals was lower than that of other developed countries. There are 18.5 physicians per 100 beds in Japan, compared to 108.1 in the UK, 93.5 in the US, 51.9 in Germany, and 51.8 in France [6]. Economists, politicians, and a group of physicians recommended reversing the physician shortage [7–9]. However, the relationship between the number of hospital physicians and the number of patient admissions in Japanese hospitals has been unclear. Thus, in the current study, we examined the association between internal medicine physician staffing and patient admissions to the department of internal medicine in teaching hospitals in Japan. Since patients with COVID-19 are included among internal medicine patients, the department of internal medicine was overwhelmed during the surge of pandemic waves.

## Methods

### Hospitals

This is an ecological study from the publicly available annual report of teaching hospitals accredited with the Japanese Society of Internal Medicine [10]. The data was collected from teaching hospitals in three prefectures (Tokyo, Osaka, Okinawa) which have experienced the highest incidence of COVID-19 during case surges in Japan. These hospitals included community hospitals and university hospitals. The community hospitals were public hospitals (including national, prefectural, city, town, village, and JCHO hospitals: JCHO = Japan Community Healthcare Organization), semi-public hospitals (Red Cross hospitals, Saiseikai hospitals, and Japan Agriculture hospitals), and private hospitals. University hospitals included the main hospitals of each university as well as affiliated (branch) hospitals. Of note, university hospitals may have interactive relationships with other community hospitals besides affiliated hospitals, due to their role of allocating doctors according to departments or divisions, which is not evaluated.

Public or semi-public hospitals are managed by public organizations, private hospitals by private organizations, and university hospitals by universities. Japan uses a system of health insurance coverage for all people and hospitals are reimbursed by the system. Based on the national standardized medical billing system, 70% of medical bills are paid by public insurance and the remainder by personal copayment or private insurance. There is a safety net system in place to reimburse the medical bill when it exceeds the limit of expensive care.

### Data collection

Data collection involved the number of admitted patients through ambulance transport per 100 beds and the number of full-time physicians per 100 beds in a one-year study period (April 2020 to March 2021). The analyzed patients were registered by the Japanese Society of Internal Medicine as patients hospitalized in internal medicine departments, and they were both COVID-19 and non-COVID-19 patients. Physicians were all related to the care of internal medicine patients, including resident physicians (postgraduate year 1–5) and staff physicians (postgraduate year 6 or higher), who were examined separately. Part-time physicians were not included in the available data, but these physicians are usually not involved in inpatient care.

### Statistical analyses

Statistical analysis was performed using STATA version 17 (College Station, NC, US). Numerical data were described as mean and standard deviation, while categorical data as percentages. Non-parametric Dunn test with post-hoc Bonferroni comparisons was used for multiple comparisons of non-normal data. Multi-variable linear regression models were constructed for examining the association between the number of physicians per 100 beds (two independent variables: resident and staff physicians) and the number of admitted patients to the department of internal medicine per 100 beds (one dependent variable). The analysis was conducted separately for community hospitals and university hospitals because the latter have roles as research institutions with tertiary care function in Japan. Statistical significance was defined as two-tailed *p*-value less than 0.05.

## Results

Of 117 teaching hospitals accredited with internal medicine specialty in three prefectures, precise data from 108 teaching hospitals (83 community hospitals and 25 university hospitals) was available for our analysis. The mean number of acute care beds was higher in university hospitals, while the mean number of ambulance transport was greater in community hospitals (Table 1). The numbers of residents and staff physicians were higher in university hospitals (Table 1).

**Table 1:** Characteristic of Community and University Hospitals ±

Characteristic (n)	Community Hospitals (*n* = 83)	University Hospitals (*n* = 25)	All Hospitals (*N* = 108)
Acute care beds, mean (SD)	456 (177)	688 (319)	510 (238)
Ambulance transports, mean (SD)	4085 (2190)	3571 (1937)	3966 (2137)
Admitted patients by ambulance, mean (SD)	1060 (609)	578 (406)	948 (602)
per 100 beds	247 (140)	94 (67)	211 (142)
Physician, mean (SD)			
Internal medicine staff physician	46 (25)	110 (84)	61 (53)
per 100 beds	10.2 (3.3)	14.5 (6.1)	11.2 (4.5)
Resident physician	32 (21)	85 (75)	44 (46)
per 100 beds	6.8 (3.3)	11.2 (6.8)	7.8 (4.7)
Staff and resident physician	78 (42)	195 (134)	105 (89)
per 100 beds	17.0 (5.6)	25.7 (9.4)	19.0 (7.6)

SD = standard deviation.

A total of 102,400 internal medicine patients were admitted to these hospitals during the one-year period. The highest admission was 3122 in a public hospital in Tokyo, while the lowest admission was 29 in a private hospital in Tokyo. Out of all patients admitted by ambulance, the proportion of patients admitted to internal medicine overall was 58.4%. The numbers were 41.9 and 62.8% in university hospitals and community hospitals, respectively.

Table 2 shows that private hospitals received the greatest mean number of patient admissions, followed by public hospitals, semi-public hospitals, and university hospitals. Patient admissions per 100 beds were not normally distributed (Shapiro-Wilk test, *p* = 0.00005). Based on the non-parametric Dunn test with post-hoc Bonferroni comparisons (Table 2), statistically significant differences were noted between university hospitals and all three types of community hospitals (private, public and semi-public hospitals).

**Table 2:** Patients Admissions per 100 beds by Hospitals (*N* = 108)

Hospital	Community Hospitals			University Hospitals
	Private Hospitals	Public Hospitals	Semi- Public Hospitals	
Thr number of hospitals	34	16	33	25
Patients admisdions per 100 beds				
mean±SD*	290±157	227±122	201±122	94 ± 67
median				
Dunn’s test *P*-value**				
Private Hospitals		0.4448	0.1793	< 0.0001
Public Hospitals			0.9999	0.0001
Semi- Public Hospitals				0.0173
University Hospitals				

*SD Standard deviation, **Multiple pairwise comparisons with Bonferronni adjustment

**Fig. 1:**
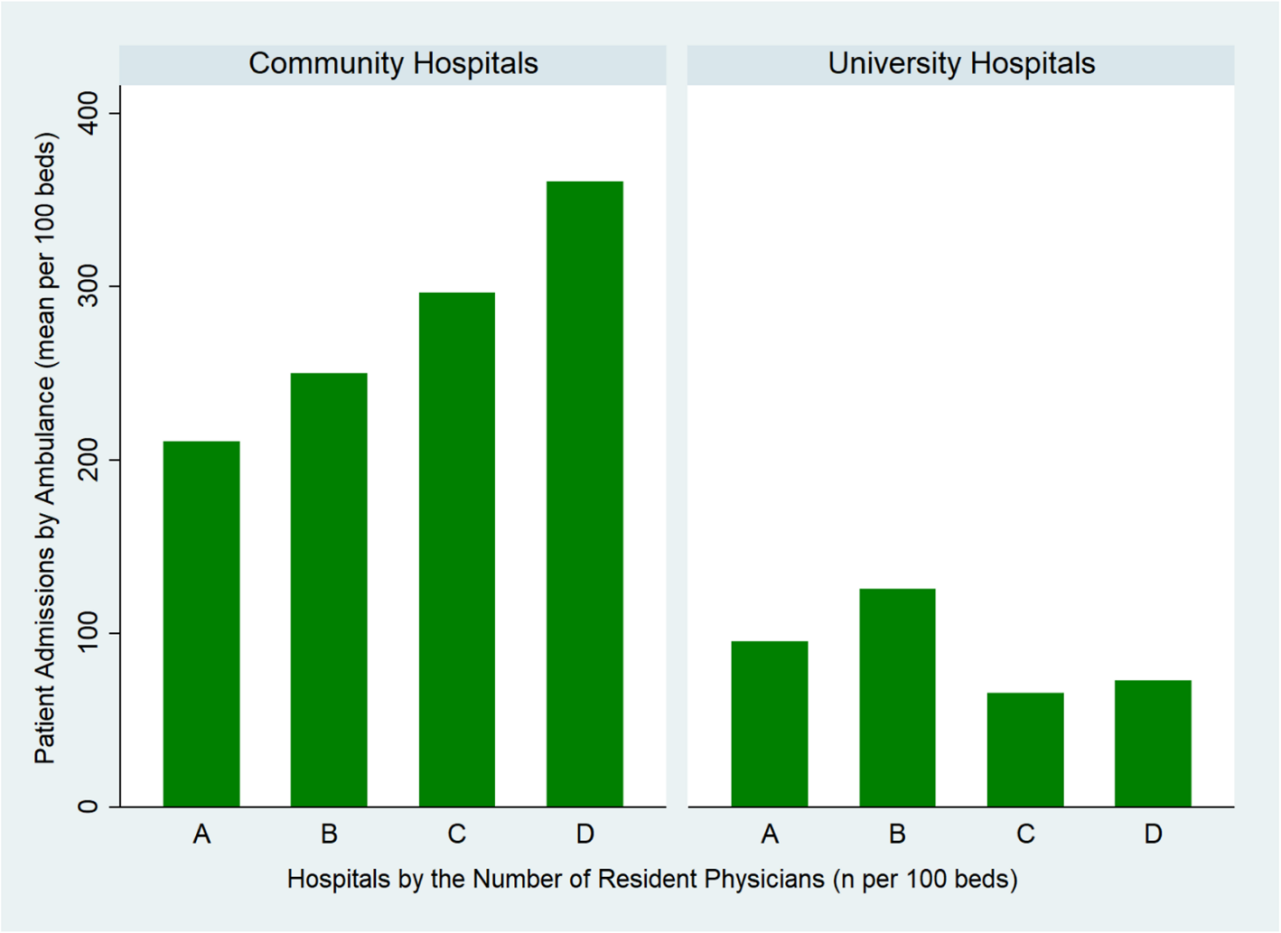
Hospitals by the number of resident physicians per 100 beds (A < 5; B > =5, < 10; C > =10, < 15; D > =15) and patient admissions by ambulance (mean per 100 beds)

Based on the multi-variable linear regression model, among community hospitals, a higher number of resident physicians per 100 beds was significantly associated with a greater number of patient admissions per 100 beds with beta coefficient of 11.6 (95% CI, 1.5 to 21.2, *p* = 0.025) admissions by one physician increase per 100 beds (Fig. 1).

**Fig. 2:**
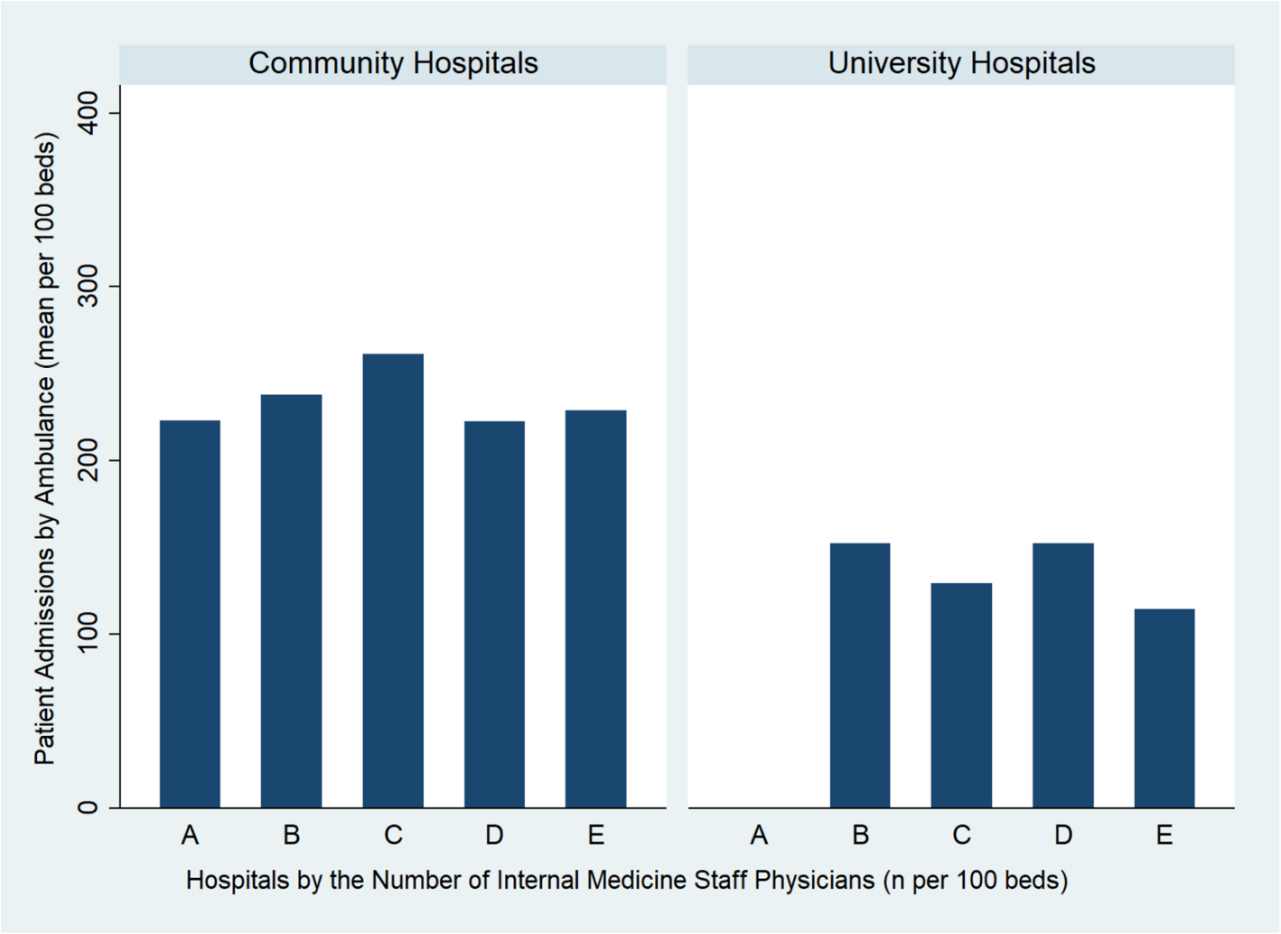
Hospitals by the number of internal medicine staff physicians per 100 beds (A < 5; B > =5, < 10; C > =10, < 15; D > =15, < 20; E > =20) and patient admissions by ambulance (mean per 100 beds)

For internal medicine staff physicians, no significant associations were noted with beta coefficient of 0.78 (95% CI, − 9.08 to 10.63, *p* = 0.876). On the other hand, in the multi-variable linear regression model among university hospitals, there was no significant association between resident physicians and patient admissions with beta coefficient of − 2.5 (95% CI, − 6.7 to 1.6, *p* = 0.221) or between internal medicine staff physicians and patient admissions with beta coefficient of − 0.29 (95% CI, − 4.98 to 4.41, *p* = 0.901) (Fig. 2).

## Discussion

In high-incidence areas of COVID-19 in Japan where physician shortage is a serious issue, community teaching hospitals with a greater resident physician workforce are significantly associated with a higher number of patient admissions. There was no such relationship among university hospitals. The number of staff physicians is also not related to admissions among both community and university hospitals. Community hospitals received more admissions per 100 beds compared to university hospitals, which was consistent with our prior hypothesis that university hospitals have roles as research institutions and highly-specialized tertiary care. Our results, at least for resident physicians, are in line with previous studies which link improvement in physician shortage with acceptance of more patient admissions [11–13]. Since Japan has the lowest number of hospital physicians by population as well as by hospital beds in OECD countries [6, 14], increasing the volume of physicians, especially resident physicians, in community hospitals should be recommended. In Japan, doctors who are 1–5 years post-graduation are classified as resident physicians. Due to their lack of experience, junior residents (1–2 years post-graduation) do not bear sole responsibility for clinical decision-making but are important members of the physician team and are expected to contribute to patient care through daily interaction with patients, medication prescription and play a substantial role in the decision-making process. Additional responsibility is given to senior residents (3–5 years post-graduation). Thus, it can be said that resident physicians play a pivotal role in the hospital setting. Although the total number of physicians has been increasing recently [15], the number of internal medicine physicians has decreased [16]. Thus, the number of internal medicine residents should be increased to meet the demands of internal medicine admissions, including COVID-19 cases with a high risk for mortality. In the long term, medical schools should accept applications from more students so as to be better prepared not only for pandemic resilience of the healthcare system but also for the increasing needs of a hyper-aging society and medical service expansion. Recent trends also show that medical schools in Japan have become increasingly popular amongst high school students, and it is difficult to enter due to the limited availability of only a small number of total slots [17]. Thus, the demand for becoming future physicians remains high for high school students in Japan and it is not difficult to recruit talented applicants.

In the short term, several strategies could be considered to enhance manpower in community hospitals. First, resident physicians’ training slots at university hospitals and other non-COVID-19 care hospitals could be transferred to community hospitals so as to be better prepared for emergencies, despite this measure being opposed to the prioritized education for medical residency. This transfer could be done as a temporary flexible measure to shift the physician workforce in midst of a pandemic wave. Second, weekend or nighttime work in community hospitals could be supported by clinic doctors during the pandemic waves if these clinic doctors have the willingness to work in community hospitals on a part-time basis. These two measures could be employed in the current health system of Japan, but investment is needed from the central and local governments to pay cooperating hospitals and clinic doctors. Third, in Japan there are nurse practitioners and medical students who are well educated to assist physicians and they may be assembled in community hospitals as temporary physician assistants in the highest incidence areas of COVID-19 in Japan. This measure calls for the development and enforcement of a new law that includes the introduction of new medical professionals such as physician assistants. Forth, introducing and enhancing telemedicine using information technology would reduce workload during the surges.

Physician workforce included staff and resident physicians, but our results revealed the significance of the number of resident physicians for accepting patient admissions. In Japan, doctors who are 1–5 years post-graduation are classified as resident physicians. Due to their lack of experience, junior residents (1–2 years post-graduation) do not bear sole responsibility for clinical decision-making and the clinical level of staff physicians is higher in knowledge and skill. However, contemporary hospital care is provided to patients by a team that includes resident physicians, and they are expected to contribute to patient care through daily interaction with patients, medication prescription and playing a substantial role in the clinical decision-making process. Additional responsibility is given to senior residents (3–5 years post-graduation). Staff physicians have roles as conductors in team care but the actual workload for admitting patients through the emergency department is mostly provided by many resident physicians based on the command of their senior physicians. Thus, it can be said that resident physicians play a pivotal role in the hospital setting. There are several limitations in the current study. First, patients in our study included all admissions to the department of internal medicine and thus non-COVID-19 patients were also included. We could not determine the disease classifications of admitted patients since our database of the Japanese Society of Internal Medicine did not include these data. However, at the time of the pandemic waves and especially during the healthcare collapse, not only COVID-19 but also non-COVID-19 patients were at risk of refusal of admission in the highest incidence areas. Thus, it may be prudent to count admissions of internal medicine patients as a health system capacity measure. Second, this research could not evaluate the following factors associated with the work contents of individual physicians; the severity of patients; research work related to COVID-19 care and the increase in efforts to teach medical students. This study was motivated to improve the Japanese healthcare system to decrease the number of refusals of necessary admissions by reconsidering the distribution of physicians who can provide care for internal medicine patients. Physicians in Japan have been noted as hard workers regardless of hospital owner organizations; we admire all the works they have done and would like to provide information which can help construct a more efficient and protective environment for them to save patients [18]. The recent enactment of The Work Style Reform Bill point towards recognition from the central government to deal with the increased workload that physicians are subjected to, and these reforms will hopefully lead to reducing the burden on these doctors hence improving the general well-being of physicians and an improved health care system. Third, the analyzed data is of one-year duration and thus cannot be compared with data from continuing years. However, if associations suggested from our results would sustain over years, we must focus on this issue with deeper concern and urgency. Forth, the cost and effect of these pandemic surge workloads among medical professionals including physicians, nurses and other co-medical staff were not assessed, besides the medical costs for COVID-19 inpatients that have been paid by national expenditure as of January 2022. Fifth, data was not available for nursing staff and other healthcare workers in the current study. These occupations are indispensable for an inpatient care team. In addition, there is no assessment of interactive or affiliated relationships between community hospitals and university hospitals in terms of dispatching workforce. Future studies are needed to examine the role of interactions of these professionals during the pandemic. In conclusion, during the COVID-19 pandemic in Japan, improving resident physician shortage may enhance patient admissions to the department of internal medicine at least in community teaching hospitals. Several measures are recommended as urgent short-term policies, including temporary flexible transfer of physician workforce to community teaching hospitals from university hospitals, non-COVID-19 care hospitals, or clinics. Nurse practitioners could become temporary physician assistants. Long-term policy change should also be considered to increase slots in medical schools.

## Data Availability

The Data is publicly available. The datasets analyzed during the current study are available in the Journal of Japanese Society of Internal Medicine (volume 110, number 12, pages 2700–2717). For the access to the datasets, please inquire the following: the Journal of the Japanese Society of Internal Medicine, The Japanese Society of Internal Medicine, 3–28-8 Hongou, Bunkyoku, Tokyo, 113–8433 Japan).
